# Assessment of Diet Quality Based on Selected Dietary Quality Indices and Consumption of Specific Food Items of Midwives Working on a Shift Schedule in Wroclaw, Poland

**DOI:** 10.3390/nu16152409

**Published:** 2024-07-25

**Authors:** Katarzyna Daria Gołąbek, Anna Chmielewska, Ewa Karoluk, Krzysztof Kujawa, Bożena Regulska-Ilow

**Affiliations:** 1 Department of Dietetics and Bromatology, Pharmacy Faculty, Wroclaw Medical University, ul. Borowska 211, 50-556 Wrocław, Poland; a.chmielewska@umw.edu.pl (A.C.); bozena.regulska-ilow@umw.edu.pl (B.R.-I.); 2 Department of Obstetrics and Gynecological and Obstetric Nursing, Health Sciences Faculty, Wroclaw Medical University, 51-618 Wrocław, Poland; ewa.karoluk@umw.edu.pl; 3 Statistical Analysis Centre, Wroclaw Medical University, 50-368 Wrocław, Poland; krzysztof.kujawa@umw.edu.pl

**Keywords:** diet, midwives, shift work, dietary habits, eating behaviour, DASH diet

## Abstract

The aim of this study was to assess the quality of diets among midwives working in a shift system and to analyze variations in their dietary habits according to their working hours. In a group of fifty midwives employed in four public hospitals in Wrocław, the HDI-2015, HEI-2015, AHEI-2010, and Mellen’s DASH diet index were calculated. The significance of differences in terms of the prevalence of selected dietary habits, meal frequency, average content of selected food items, and the percentage of energy obtained from them was assessed. Over half of the diets of the participants exhibited low adherence to the selected dietary indices. Only the scores on Mellen’s DASH diet index were significantly associated with other components of the diet. Diets scoring ≥ 4.5 points were characterized by significantly lower processed meat content, meal frequency, and energy value, as well as lower sugar content and lower dietary energy value, compared to diets scoring < 4.5 points. Regardless of their working hours, the diets of midwives are characterized by low quality. Therefore, it appears essential to introduce targeted educational programs and provide guidance on appropriate dietary models, such as the DASH diet.

## 1. Introduction

Shift work, due to the extended duration of nighttime activity, can disrupt the biological clock function [1]. Therefore, this type of work, especially over an extended period of time, may increase the risk of cardiovascular issues [2,3], colorectal cancer [4], or type 2 diabetes (T2D) [5]. It appears that women are particularly vulnerable to the adverse effects of such work on their health. Some research results imply that prolonged exposure to shift work in women may be associated with a higher risk of obesity [6].

Moreover, shift work not only impacts directly on human health. Some reports of shortened sleep duration and poor sleep quality showed also a correlation with lower diet quality [7,8]. Shift work encourages the development of unhealthy dietary habits, such as snacking [9] and stress-induced eating [10]. It was found that working night shifts led to increased consumption of ready-to-eat fried food and sweetened beverages [11]. Also, a significantly higher prevalence of “sugar-sweetened drinkers” (high intakes of juice/non-alcoholic beer and beverages and “low” for water) among shift workers in comparison to day workers was observed [12]. In other studies, researchers emphasized high sugar consumption in shift workers [13], particularly during night shifts [14].

There are a number of female healthcare professionals who often work in shifts. Despite their knowledge of proper nutrition, it does not always manifest itself in their dietary habits [8]. It is noted that midwives belong to a group of healthcare workers of particular importance in shaping a healthy lifestyle due to their contact with pregnant women and newborn mothers. Midwives are listed as key healthcare professionals who are responsible for providing antenatal care in most countries. It is crucial for them to build and enhance their ability to promote healthy lifestyles in pregnant women [15].

The assessment of diet based on specific dietary quality indices (DQIs) is an essential tool that enables comparisons of dietary choices between different populations [16]. Researchers assess the diet quality of a selected group using various indices based on different recommendations to determine a potential link between the pattern of dietary habits and an increased risk of lifestyle-related diseases in a given population [17].

The scores of the selected indices correlate with the risk of diseases aggravated by the occurrence and/or extension of shift work. For example, in a group of over 12,000 participants, higher Health Eating Index—2010 (HEI-2010), Alternative Healthy Eating Index—2010 (AHEI-2010), and DASH scores were associated with a lower risk of all-cause mortality, cardiovascular-related deaths, and cancer-related deaths [18]. This is of particular relevance given the findings to date, according to which the incidence of cardiovascular incidents is positively associated with shift work. A literature review and meta-analysis of observational studies found a higher risk of myocardial infarction and coronary incidents among shift workers [19]. When considering shift work as an independent risk factor, midwives with poor-quality diets who perform shift work may be at significant risk of cardiovascular disease. In another study, higher scores on the AHEI-2010 were linked to a lower risk of overall mortality, cardiovascular-related deaths, and inflammatory-related deaths in postmenopausal women [20]. The DASH diet has been a significant dietary pattern for both healthy individuals and those with various disease entities such as obesity, hypertension, and hepatic steatosis [21,22]. In the available literature, many studies propose proprietary indices for assessing adherence to the DASH diet recommendations. For example, in the study by Miller et al. [23], it was assessed that three different indices by Mellen [24], Fung [25], and Günterr [26] correlated at similar levels with the risk of colorectal cancer in women. In the dietary pattern by Mellen et al. [24], in contrast to the other two, instead of assessing the number of portions of specific foods in the diet, the nutrient content of the diet or the percentage of energy (%E) derived from macronutrients was assessed. 

Indices used in clinical research are updated to adapt to current dietary guidelines. The HEI-2015 was modified to align with the updated dietary guidelines for the North American population from 2015 to 2020 [27]. Low scores on the HEI-2015, similar to its previous versions, are associated with an increased risk of overall mortality, cardiovascular-related deaths, and cancer-related deaths [28]. Similarly, in 2018, the Healthy Diet Indicator (HDI)—promoted by the WHO as a tool to assess the healthiness of the diet—was updated [29]. It appears that analysis of diets using several different indicators with confirmed correlation with women’s health allows for a comparison of diets to various nutritional models with health-promoting effects.

This study aims to assess the quality of the diets of shift midwives based on four selected DQIs describing healthy diets: the HEI-2015, HDI-2015, AHEI-2015, and Mellen’s DASH diet index. This work complements previous analyses of nutrient content in the diets of this population [30]. Another aim of this study is to analyze the dietary habits of midwives and asses their variations according to working hours. This study aims to determine which elements of the shift schedule (day shift, night shift, or non-working day) might affect the deterioration of dietary habits.

## 2. Materials and Methods

**Figure 1:**
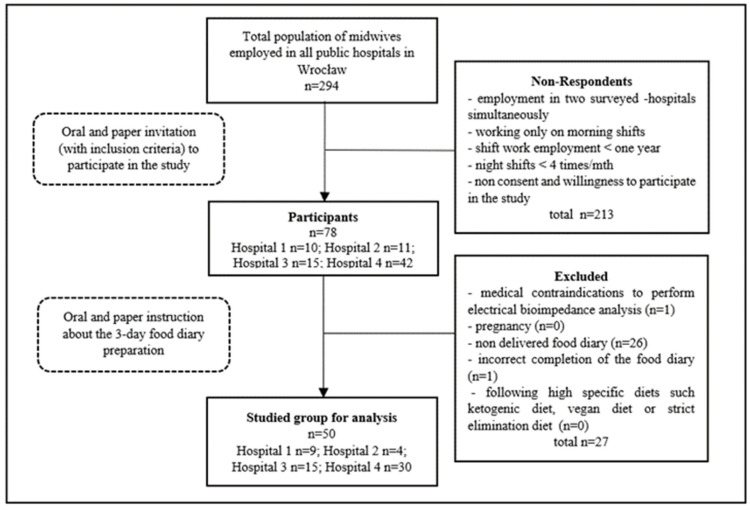
Flowchart of the study recruitment process.

The study group consisted of 50 midwives who fully completed this study. To obtain a homogenous group, only midwives working in one city were invited to participate. All midwives employed in all 4 public hospitals in Wrocław, the 3rd most populous city in Poland, were requested to join the survey. Figure 1 presents the recruitment process of the study group.

In this study, shift schedule was defined mainly as consecutive days with a morning shift (working hours 7:00–19:00), a night shift (19:00–7:00), and two days off.

This study was approved by the Ethics Committee of the Wroclaw Medical University.

To assess the nutritional status of the participants (*n* = 78), anthropometric measurements such as height, weight, body composition by bioelectrical impedance analysis, and waist and hip circumference were conducted. To measure height, a free-standing, portable TANITA HR-001 (TANITA, Tokyo, Japan) stadiometer was used. Body composition and weight were performed by a TANITA BC 545 (TANITA, Tokyo, Japan) bioelectrical impedance analyzer. Waist and hip circumference were measured using a standard measuring tape with an accuracy of 1 cm. Waist circumference was measured halfway between the top of the hip bone and the lower rib curve. Hip circumference was measured at the widest point below the wings of the ilium, at the bulge of the buttocks. BMI (Body Mass Index) values were calculated by the equation BMI = weight (kg)/height (m^2^), and a range of 18.5 to 24.9 kg/m^2^ was considered normal [31,32].

The prevalence of nutritional habits and quality of the study participants’ diets were assessed by a 3-day food diary (*n* = 50). The food diary record included 1 shift day, 1 night shift day, and 1 non-working day. The participants were asked to self-record their typical daily diet and were instructed not to choose unusual days with significantly different food choices, such as parties or holidays. At the beginning of this study, midwives were informed on how to correctly record their food and drink intake and how to assess the portions by dietitians. Also, they had written instructions in the paper 3 day food diary prepared by the authors. 

The mass of consumed food, expressed in household measures, was specified with the use of an “Album of photographs of food products and dishes” [33]. The computer program “Food Processor” SQL version 9.8.1’s ESHA Research (USA), which contains a Polish database of products and dishes, was used to analyze the dietary data [34]. Based on the data obtained, the macronutrient content and the percentage of energy from each one in the daily ration were calculated. Also, the content of selected micronutrients (vitamins A, D, E, C, B1, B2, B6, and B12 and folic acid, minerals, calcium, iron, copper, phosphorus, magnesium, zinc, iodine, sodium, potassium) were determined.

Furthermore, based on a 3-day food diary, the average consumption of selected food items was assessed, including added sugar, sweetened beverages, sweets and pastries, caffeine, processed meat, vegetables, fruit, fish, nuts and seeds, natural dairy products, and pulses. Additionally, the number of meals and their energy value were assessed. The average caffeine intake was calculated based on recorded consumption from dietary sources, such as coffee, tea, caffeinated soft drinks, cocoa, and chocolate products. This was performed by considering the average caffeine content in these products from popular brands available in Poland [35]. 

The average energy value and nutritional value of each participant’s diet were assessed based on data from product and meal consumption recorded in a 3-day diary. Diet quality was assessed using the following indices, with the scoring criteria presented in Table 1, Table 2, Table 3 and Table 4.

(1) Healthy Diet Indicator—2015 (HDI-2015) [29]

**Table 1:** Scoring criteria for the Healthy Diet Indicator—2015.

	Healthy Diet Indicator—2015			
	Component	Unit	1 Point	0 Points
1.	Daily avg. fruit and vegetable consumption	[g]	>400	<400
2.	%E from fat	[%]	<30	>30
3.	%E from SFAs	[%]	<10	>10
4.	%E from PUFAs	[%]	6–11	<6 or >11
5.	%E from simple sugars *	[%]	<10	>10
6.	Daily fiber intake	[g]	>25	<25
7.	Daily potassium content	[mg/d]	≥3500	<3500
			**max. total score—7 points**	**min. total score—0 points**

E—total daily energy intake, SFAs—saturated fatty acids, PUFAs—polyunsaturated fatty acids. * Free sugars were defined as simple sugars (glucose, sucrose, maltose) added by manufacturers to food products (sweets, sweetened beverages, confectionery products), simple sugars were those that were added by consumers during sweetening of dishes and those naturally occurring in honey, syrups, and fruit juices [36].

High adherence to a healthy diet was considered with scores of 6–7 points, moderate adherence with 4–5 points, and low adherence with 0–3 points.

(2) DASH Diet Index by Mellen et al. [24]

**Table 2:** Scoring criteria for Mellen’s DASH diet index.

	DASH Diet Index by Mellen et al. [24]				
	Component	Unit	1 Point	0.5 Points	0 Points
1	%E from protein	[%]	≥18	<18 to ≥16.5	<16.5
2	Dietary fiber/1000 kcal of the diet	[g]	≥14.8	<14.8 to ≥9.5	<9.5
3	Mg/ 1000 kcal of the diet	[mg]	≥238	<238 to ≥158	<158
4	Ca/1000 kcal of the diet	[mg]	≥590	<590 to ≥402	<402
5	K/1000 kcal of the diet	[mg]	≥2230	<2230 to ≥1534	<1534
6	%E from fat	[%]	≤27	>27 to ≤32	>32
7	%E from SFAs	[%]	≤6	>6 to ≤11	>11
8	Cholesterol/1000 kcal of the diet	[mg]	≤71.4	>71.4 to ≤107.1	>107.1
9	Na/1000 kcal of the diet	[mg]	≤1143	>1143 to ≤1286	>1286
			**max. total score—9 points**		**min. total score—0 points**

E—total daily energy intake; SFAs—saturated fatty acids; diets scoring above 4.5 points were considered consistent with the DASH diet.

(3) Healthy Eating Index—2015 (HEI-2015) [27,37]:

**Table 3:** Scoring criteria for the Healthy Eating Index—2015.

	Healthy Eating Index—2015					
	Component	Unit	Max. Score per Section	Criteria for Max. Score	Min. Score per Section	Criteria for Min. Score
1.	Total fruits	c equivalents/1000 kcal	5	≥0.8	0	0
2.	Whole fruits	c equivalents/1000 kcal	5	≥0.4	0	0
3.	Total vegetables	c equivalents/1000 kcal	5	≥1.1	0	0
4.	Green vegetables and pulses	c equivalents/1000 kcal	5	≥0.2	0	0
5.	Whole-grain foods	oz equivalents/1000 kcal	10	≥1.5	0	0
6.	Low-fat dairy products	c equivalents/1000 kcal	10	≥1.3	0	0
7.	Low-fat protein products (chicken, turkey, veal, rabbit) and eggs	oz equivalents/1000 kcal	5	≥2.5	0	0
8.	Protein products of marine and plant origin	c equivalents/1000 kcal	5	≥0.8	0	0
9.	Ratio: %E from (PUFAs +MUFAs)/%E from SFAs		10	≥2.5	0	≤1.2
10.	Refined grains	oz equivalents/1000 kcal	10	≤1.8	0	≥4.3
11.	Na/1000 kcal of the diet	[g]	10	≤1.1	0	≥2.0
12.	%E from added sugars	[%]	10	≤6.5	0	≥26
13.	%E from SFAs	[%]	10	≤8	0	≥16
			**max. total score—100 points**		**min. total score—0 points**	

E—total daily energy intake, SFAs—saturated fatty acids; MUFAs—monounsaturated fatty acids; PUFAs—polyunsaturated fatty acids. The equivalents of portions of food products were estimated based on the My Pyramid Equivalents Database [38], while the quantity and quality of ingredients in Polish dishes were estimated based on recipes found in tables of food composition and nutritional value [34].

Scores of 90–100 points were considered extremely high adherence to the dietary pattern described by the HEI-2015; 80–89 points were considered high, 70–79 points good, 60–69 points moderate, and < 59 points low adherence [39].

(4) Alternative Healthy Eating Index—2010 (AHEI-2010) [40]

**Table 4:** Scoring criteria for the Alternative Healthy Eating Index—2010.

	Alternative Healthy Eating Index—2010					
	Component	Unit	Max. Score per Section	Criteria for Max. Score	Min. Score per Section	Criteria for Min. Score
1.	Total vegetables	[s/d]	10	≥5	0	0
2.	Total fruits	[s/d]	10	≥4	0	0
3.	Whole grains	[g/d]	10	75	0	0
4.	Sugar-sweetened beverages and fruit juices	[s/d]	10	0	0	≥1
5.	Nuts and legumes	[s/d]	10	≥1	0	0
6.	Red/processed meat	[s/d]	10	0	0	≥1.5
7.	%E from TRANS FATS	[%]	10	≤0.5	0	≥4
8.	EPAs + DHAs	[mg/d]	10	250	0	0
9.	%E from PUFAs	[%]	10	≥10	0	≤2
10.	Sodium content	[mg/d]	10	<1279.47 **	0	>2840.26 ***
11.	Alcohol	[drinks/d]	10	0.5–1.5 *	0	≥2.5
			**max. total score—110 points**		**min. total score—0 points**	

E—total daily energy intake; EPAs—eicosapentaenoic fatty acids; DHAs—docosahexaenoic fatty acids; PUFAs—polyunsaturated fatty acids. The equivalents of portions of food products were estimated based on the My Pyramid Equivalents Database [38], while the quantity and quality of ingredients in Polish dishes were estimated based on recipes found in tables of food composition and nutritional value [34]; *—additional criteria 2.5 points for 0 drinks/d; **—lowest decile of the studied population, ***—highest decile of the studied population.

A score of 2.5 points was assigned to non-drinkers following the approach of Chiuwe et al. [37]. Similar to the HDI-2015, adherence to the AHEI-2010 was classified as follows: 110–100 points indicated perfect adherence, 90–100 points indicated extremely high adherence, 80–89 points indicated high adherence, 70–79 points indicated good adherence, 60–69 points indicated moderate adherence, and <59 points indicated low adherence.

Moreover, the researchers assessed the significance of differences in the prevalence of selected dietary habits, such as sweetening beverages, snacking between meals and sweets (including cakes and confectionery products), processed meat, salty snacks, natural dairy products, nuts and seeds, pulses, and fish consumption. The use of whole-grain cereal products was also assessed. 

Additionally, the researchers looked at the average consumption of selected food products and the percentage of energy obtained from them, categorizing the data into two groups based on correct values of selected anthropometric indices (measurements). The age division (≤50, >50 years) was based on reports on the average menopausal age in Poland and Eastern Europe. Mostly, the average menopausal age was assessed to be above 50 years of life (Mdn—51.25 [41], Mdn—52 [42], M—51.3—[43]), which was taken as the cut off.

The BMI cut-off values (<25 kg/m^2^, ≥25 kg/m^2^) were determined by the WHO recommendation for the European population [31], which was to divide the participants into groups with proper and excessive body weight (overweight and obesity). Similarly, the waist circumference cut off (≤80 cm and >80 cm) was based on the International Diabetes Federation recommendation and the WHO recommendation for increased risk of metabolic complications [31,32]. 

The body fat (BF) content cut off was established at 30%. The participants were purposely divided into groups with proper and excess body fat content (overweight and obesity). The authors adopted the AACE/ACE guideline, which assumed obesity in women > 35% BF, and reduced it to 30% to obtain the value referred to as the proper weight [44]. It is reflected in reports from a Macek et al. study [45]. In this Polish women study group (*n* = 3145), the high prevalence (87.2%) of one or more cardiovascular risk factors (CRFs), such as dyslipidemia, diabetes mellitus, unhealthy diet, smoking, physical inactivity, and family history, among participants with 30–35% BF, was observed. Such a high level of CRF was not noted in a group with lover body fat content. Moreover, a dynamic increase in hypertension prevalence and more than two other CRFs among women with 30% BF was assessed.

Furthermore, the researchers used the percentage of daily energy intake obtained from snacking (≤13%E, >13%E), the percentage of daily energy intake obtained from sweets (≤12%E, >12%E), and the consumption of red processed meat (≤50 g/d, >50 g/d) as criteria for division. The cut-off values for energy from snacking and sweets were determined by the population’s average consumption. Based on reports that every 50 g of processed meat eaten daily increases the risk of colorectal cancer by about 18% [46], the authors established that amount of processed meat as a division criterion. 

Moreover, the researchers assessed the significance of differences in selected anthropometric measurements, the prevalence of selected dietary habits, the average quantities of selected food products, and the %energy obtained from their consumption in two groups based on adherence to diets described by the following indices: the DASH diet index (≥4.5 points <4.5 points), HDI-2015 (≥4 points, <4 points), HEI-2015, and AHEI-2010 (≥60 points, <60 points).

### Statistical Analysis

In the statistical analysis of the collected data, the researchers compared and assessed the significance of differences in the frequency and quantity of consumption of the selected food groups, the number of meals, the frequency of snacking, and the percentage of energy obtained from snacking on morning shift days, night shift days, and non-working days. 

The statistical analysis was conducted using the “Statistica v 12.0 PL” software from StatSof Inc., Tulsa, OK, USA. 

The PERMANOVA test was used to check the effect of the hospital on the studied women’s features, and thus, to check whether the sample was homogeneous. First, the assumption of the PERMANOVA was checked using a permutation test for homogeneity of multivariate dispersion. Both tests were performed using 999 permutations (default values) without any constraints and with the aid of the “vegan” R package [47].

A permutation test for homogeneity of multivariate dispersions is as follows: F = 0.17, *df* = 3, *p* = 0.919.

PERMANOVA: F = 1.27, *df* = 3, *p* = 0.271. 

The test results prove that no clustering effect of the hospital has occurred, and the sample used in this study can be considered homogeneous.

The significance of differences in habit changes between morning shift days, night shift days, and non-working days was assessed using the McNemar test. The significance of quantitative differences between morning shift days, night shift days, and non-working days was estimated using the non-parametric Friedman test for repeated measures. If there was *p* < 0.05 in this test, a Wilcoxon post hoc test was performed for pairs related to the Bonferroni correction. 

The significance of the differences between the two groups was assessed based on a *t*-test in the case of a normal distribution, as determined by the Shapiro–Wilk test. The Mann–Whitney test was used in the absence of a normal distribution. A significance level of *p* < 0.05 was adopted for this study.

## 3. Results

### 3.1. The Characterization of the Study Group

A total of 78 caucasian midwives began this study, and 50 of them fully completed it (a response rate of approx. 65%). The mean age of the subjects was 45 ± 10.9 years (min—25 years; max—70 years).

The studied women were employed in the following departments: gynecology-obstetrics (22%), neonatology (6%), delivery room (26%), neonatal intensive care and emergency room (4%), and pregnancy pathology (38%).

A total of 98% of the study participants followed the omnivorous diet. Only one person limited dairy products (but used butter) and limited dietary sources of gluten, without medical indications.

A total of 20% of the participants used pharmacological treatment to improve cardiovascular health. A total of 18% of the women took antihypertensives, 2% took antihyperlipidemic drugs, and 4% took medication for varicose veins. A total of 16% of the midwives had hypothyroidism treatment. A total of 4% of the participants used hormone replacement therapy, 4% used oral anti-diabetic drugs, 6% used antiallergic drugs, 4% used anti-inflammatory medications, and 4% used PPI.

Based on a 3-day food diary, only 8% of the studied women consumed alcohol (no one daily).

### 3.2. DQIs

#### 3.2.1. HDI-2015

According to the data in Table 5, which presents the percentage of analyzed diets with a different level of adherence to the HDI-2015, sixty-six percent (66%) of the diets of the surveyed women revealed a low consistency with a healthy diet, according to the 2015 WHO recommendations. The majority of the participants achieved the recommended daily intake of vegetables and fruit > 400 g/day (66%), had a lower than 10%E contribution from free sugars (70%), and maintained a moderate potassium content (≥3500 mg/day) (46%). The fewest participants adhered to the guidelines regarding the percentage of energy derived from total fat < 30%E (30%), %E from SFAs < 10%E (20%), and %E from PUFAs (6–11%) (28%).

#### 3.2.2. Mellen’s DASH Diet Index

Based on the selected index, it was estimated that 40% of the analyzed diets conformed to the DASH diet. The mean score was 3.51 ± 1.7 (*n* = 50). The highest scores were related to adequate potassium content (58% of diets) and the lowest to cholesterol content (10% of diets) and %E from SFAs (15% of diets).

#### 3.2.3. HEI-2015 and AHEI-2010

**Figure 2:**
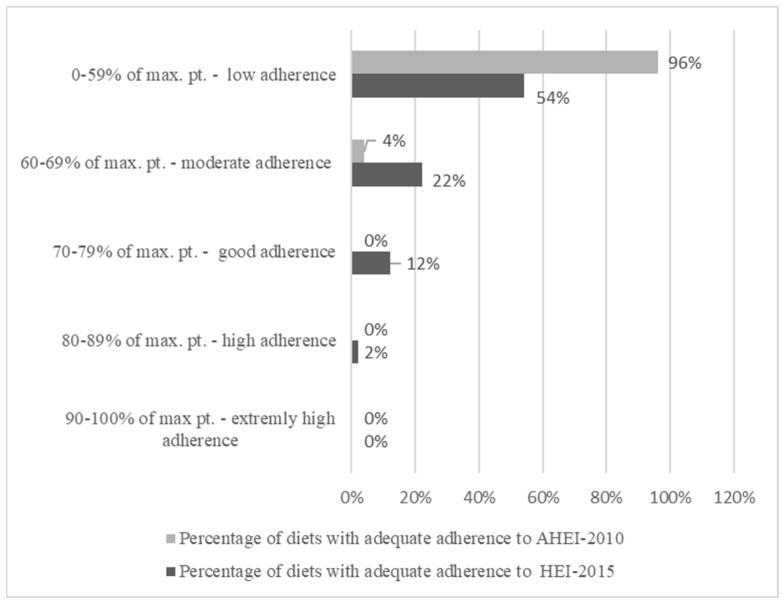
Percentage of diets with adequate adherence to the dietary patterns described by the HEI-2015 and AHEI-2010 indicators.

Figure 2 presents the comparison of the adherence to the HEI-2015 and AHEI-2010 indicators. The mean HEI-2015 value estimated for the diets of the surveyed midwives was approximately 56 points (55.79 ± 12.43). The minimum value was set at 29.55 points, and the maximum was at 81.93 points. The fewest points were awarded in relation to the content of green vegetables and pulses and the content of marine protein products and pulses. In contrast, the highest scores were obtained for a normal sodium/1000 kcal diet with %E from added sugars and the intake of whole-grain foods.

The mean scores on the AHEI-2010 of the diets of the surveyed women were approximately 39 points (38.62 ± 7.68). The minimum value was assessed at approximately 27 points, and the maximum value was at 62 points. The analyzed diets scored lowest for the intake of nuts and alcohol and the highest for the absence of trans fatty acids, fruit juices, and sweetened beverages. 

Table 6 presents a summary of the percentages of diets that demonstrate appropriately indicated adherence to more than one of the tested indicators. Only 16% of the diets of the surveyed women were consistent with the reference diet as assessed by three indices at a level at least greater than low (omitting the AHEI-2010). Similarly, only 20% of the diets simultaneously achieved a higher than low level of adherence to both the DASH diet and the dietary pattern described by the HDI-2015.

Out of all the indices used, only the scores on Mellen’s DASH diet index were significantly associated with other dietary components that are not assessed for the index calculation. 

Table 7 shows a comparison of the values of anthropometric measurements and dietary components in the two groups of participants, depending on their Mellen’s DASH diet index score. 

Diets with scores ≥ 4.5 points were estimated to have a significantly lower processed meat content, a lower number of meals, and a lower energy value for breakfast compared to diets with scores < 4.5 points.

In addition, diets that are consistent with the DASH diet contained significantly less sugar and had a significantly lower proportion of total energy and energy derived from the consumption of sweets compared to diets with low adherence to the index used.

### 3.3. Number of Meals

**Figure 3:**
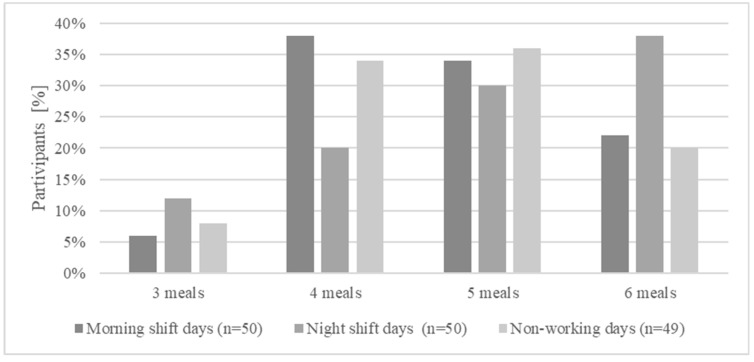
The percentage of surveyed women consuming a specific number of meals by working day.

The surveyed women consumed 4–5 meals ($\bar{X}$ ± SD 4.78 ± 0.94) per day. However, the number of meals did not significantly differ between the morning shift, night shift, and non-working day, which is presented in Figure 3.

The most common meals missed out of the standard five were supper, afternoon snack, and brunch. Although all surveyed women consumed breakfast daily, in approximately 15% of cases, its energy value was less than 100 kcal, and the breakfast generally consisted of coffee with milk and sugar. 

Although the surveyed women consumed an appropriate number of meals, approximately 70% of them snacked between meals each day and, on average, obtained approximately 13% of their dietary energy value from snacking. The percentages of energy obtained from snacking ranged from 0%E (minimum) to 46.72%E (maximum). 

Table 8 shows a comparison of the values of anthropometric measurements and dietary components in the two study groups according to the percentage of energy obtained from snacking, which were both below and above the average for the entire study group—13%.

### 3.4. Consumption of Selected Product Groups

On average, approximately 56% of the surveyed women included sweets in their diet. The surveyed women consumed cakes, confectionery products, desserts, and chocolate products, obtaining, on average, approximately 11.55% of E from these sources. It should be noted that the percentages of energy derived from these sources varied widely, ranging from 0% to 70.1% of E. The consumption of sweets did not significantly differ based on working hours. 

Table 9 shows a comparison of the values of anthropometric measurements and dietary components in the two study groups according to the percentage of energy obtained from the consumption of sweets, which were both below and above the average for the entire study group—12%.

Diets in which the participants obtained ≤12% of their E from sweets were marked by significantly lower energy values compared to diets obtaining >12% of their E from these products. However, participants with diets with higher energy intake from sweets were marked by significantly smaller waist circumferences and lower body fat content compared to those reporting lower sweet consumption.

The women whose diets provided ≥12% of their E from consuming sweets significantly and more frequently consumed refined carbohydrate sources, and in their diets, whole-grain foods constituted a maximum of 25% of the used carbohydrate sources throughout the day compared to women who consumed fewer sweets (Mann–Whitney U test, U = 185, Z = 2.267, *p* = 0.023). 

In this study, however, it was assessed that women with body fat content ≤ 30% (*n* = 15) had significantly lower BMI values (21.03 ± 11.50; M—20.68 vs. 29.24 ± 4.41 M—29.06; Mann–Whitney U test, U = 9.0, Z = −5.356, *p* < 0.001). Still, they obtained significantly higher percentages of their E from consuming sweets compared to women with a higher body fat content (*n* = 35).

Table 10 shows a comparison of the dietary components in the two study groups according to the participants’ body fat content.

Similarly, it was assessed that women with a waist circumference ≤ 80 cm (*n* = 18) more often consumed sweets over three days and obtained higher energy values from their consumption. They also obtained higher energy values in their diets compared to women with a waist circumference > 80 cm.

Table 11 shows the comparison of dietary components in the two study groups according to the women’s waist circumference values.

Forty-four percent (44%) of the participants used sugar to sweeten their beverages on a daily basis, increasing their intake of simple sugars.

The surveyed women less frequently chose salty snacks, such as crisps, salty sticks, popcorn, or crispy-coated peanuts, from which they obtained an average of approximately 1% of their E intake. 

Table 12 shows the mean content of key food items in the assessment of diet healthiness, such as added sugar, sweets, and sweetened beverages, as well as vegetables, fruit, natural dairy products, and whole-grain foods.

#### 3.4.1. Sources of Protein

The participants, on average, consumed approximately 53 ± 59.34 g of processed meat per day. Approximately 64% of them included such products in their diet. A significantly higher number of women chose processed meat during the morning shift compared to the night shift (McNemar’s chi square (*n* = 49, *df* = 1) = 5.063, *p* = 0.024). On average, the surveyed women obtained approximately 5% of their E from such products (from min. 0 to max. 28.1%E). 

Most surveyed women (>80%) consumed natural dairy products on a daily basis and, on average, 30% of them additionally included eggs. Approximately 12% of the participants included fish as part of their diet. 

#### 3.4.2. Vegetables and Fruit

Diets of women contained, on average, approximately 254.40 ± 234.48 g of fruit and 442.5 ± 234.99 g of vegetables. 

#### 3.4.3. Bakery Products

The majority of the surveyed women chose bread and bakery products made from refined flours, as well as confectionery products, as their primary sources of complex carbohydrates in their diets. On average, 32% of the assessed diets traditionally included whole-grain foods (constituting 75% of all cereal products consumed during the day). 

#### 3.4.4. Caffeine and Beverages

The average daily caffeine intake was estimated at an average of 147.27 ± 100.89 mg, which corresponds to the daily permissible consumption for healthy individuals. The surveyed women very rarely chose juices, fizzy drinks, or alcohol as part of their diet. 

#### 3.4.5. Other Products

The surveyed women rarely and in small quantities consumed highly nutritious products such as pulses, dried fruit, nuts, and seeds. The content in the diets of the surveyed women was approximately 5–7 g per day (7 g of nuts and seeds, 5 g of dried fruit, 5 g of pulses), and they were chosen by 14–18% of the participants as part of their diets.

## 4. Discussion

Although the mean vitamin and mineral contents of the participants’ diets mostly met the requirement (with the exception of vitamin D, iodine, and calcium) and the mean energy structure of the diets did not differ significantly from that recommended for healthy individuals [30], the analysis of DQIs revealed the poor quality of the midwives’ diets. The occurrence of studied dietary habits, such as sweetening beverages, number of meals, snacking, and sweets intake, was not significantly different between shift working hours. Only differences in processed meat intake between the morning and night shifts were observed. The presence of unhealthy habits was more a repeatable daily pattern than a constant behavior connected only with specific working hours (night shift, morning shift, or non-working day). 

In this study, the diet quality of midwives working in a shift system was assessed using four different dietary indices: the HDI- 2015, DASH diet index, HEI-2015, and AHEI-2010, in which low adherence to the dietary model presented in the indices was assessed in 66%, 60%, 54%, and 96% of midwives’ diets, respectively. It appears that the use of indices enables a more precise assessment of the quality of diets than the average content of nutrients. The quality indices assess the diet as a dietary model that is composed of several, rather than one, strategies for health maintenance and nutritious components. 

However, each of the analyzed dietary indicators classifies diet quality based on different criteria. The large discrepancy in the percentage of surveyed diets, which were characterized as low quality, resulted from differences in the nutritious components and their number of awarding points. The DASH diet index is based on the supply of selected dietary components, while the other selected indicators award points in terms of both the consumption of selected product groups and the nutrient supply. However, using four indicators for quality assessment allows for a wider and multiaspect evaluation of dietary choices and habits in the studied population.

In a meta-analysis involving 68 studies with 1,670,179 participants compiled by Schwingshackl et al. [48], diets that scored high on different types of HEIs (HEI, HEI-2005, HEI-2010), AHEIs (AHEI, AHEI-2010), and three versions of the DASH index were associated with a significant reduction in the risk of all-cause mortality (22%), cardiovascular diseases (22%), cancers including breast, prostate, colorectal, and other cancers (16%), T2D (18%), and neurodegenerative diseases, such as Parkinson’s disease (15%). In a study involving Iranian nurses, there was an inverse correlation between adherence to the DASH diet and the prevalence of obesity. Those in the highest quartile of the DASH diet score were 71% less likely to have general obesity compared to those in the lowest quartile. Furthermore, adherence to the DASH diet was inversely associated with the prevalence of central obesity after potential confounders were taken into consideration. Adherence to the DASH diet was also associated with significantly higher intakes of fruits, vegetables, low-fat whole-grain foods, nuts, and pulses [49].

In three large prospective cohorts among the study population from the following studies, the Nurses’ Health Study, Nurses’ Health Study II, and Health Professionals Follow-up Study, greater adherence to health-promoting dietary patterns—including the HEI-2015 and AHEI-2010 analyzed in this study—was associated with a lower risk of cardiovascular diseases [50]. In population-based analyses from the above-mentioned studies, correlations between dietary scores and cardiovascular disease risk were consistent across all racial/ethnic subgroups, regardless of body weight, physical activity, smoking, alcohol consumption, marital status, and family history of cardiovascular diseases.

Due to the mentioned outcomes, it is worth emphasizing that only max. 22% of the studied diets achieved moderated or higher adherence to two, 16% to three, and only 4% to four indices simultaneously. Expected good or high adherence to these indices, associated with better health and lower risk of diseases, was achieved by max. 8% of diets to two indices, 4% to three, and none to four.

According to DQIs, the midwives from our study scored lowest for the dietary fat and cholesterol intake and percentage of saturated fatty acids in the total daily rations. A high dietary fat intake was also reported in other studies involving Polish midwives [51,52]. Nurses and midwives often work long and irregular hours in high-stress environments, likely contributing to high rates of burnout. Shift workers need to adopt psychological methods of coping with stress to cope with the sleep and health challenges that are associated with an irregular work schedule. In a study evaluating the relationship between stress coping styles and dietary intake among shift nurses and midwives, it was found that the recognition of wishful thinking and withdrawal among female employees was associated with a higher daily intake of energy, total fat, polyunsaturated fat, monounsaturated fat, carbohydrates, sugar, and cholesterol [53].

In the study by Beebe et al. [54], both night shift nurses and day shift nurses had low-quality diets in terms of fatty acids, whole-grain foods, and sodium intake. In this study, although the midwives’ diets scored high in terms of correct sodium content, it should be mentioned that the dietary analysis included sodium contained in the products consumed without taking into consideration the salting of food after preparation, “on the plate”, which may have significantly influenced the final dietary intake of this element. This, in turn, could contribute to the higher scores in the sodium content aspect of the dietary indices.

The diets of midwives in this study were also distinguished by a low content of pulses, nuts, fish, and green vegetables and a high content of sweets and confectionery products compared to the recommended values considered in the indices analyzed. Despite working in healthcare, the nature of which would suggest greater knowledge of the role of diet and a balanced lifestyle in maintaining health, their diets do not differ compared to the standard diet of Poles [55]. In the national report on diet and nutritional status, women aged 18–65 declared consuming pulses several times a month (29.5%). More than 1 in 10 participants did not include products from this group in their diet. Another food group, leafy green vegetables, was most commonly consumed 2–3 times a week by 36.2% of women.

In this study, the midwives consumed 4–5 meals per day, regardless of the work shift worked. This is a higher number of meals compared to the average number declared by Polish women aged 18–64. In this group, the highest percentage of participants declared eating three meals a day (49.2% of women). In contrast, four meals were consumed by 31.1% of them. Approximately 45% of the women did not consume meals at fixed times. In this study, approximately 70% of the midwives declared that they ate between meals each day, and the percentage of energy gained from eating reached almost 50% of the dietary energy. In a study by Beebe et al. [54], 82.7% of the surveyed nurses who worked night shifts declared snacking at least once a day.

In a study by Lin et al. [56] involving shift nurses, energy intake in estimated energy requirements was significantly lower during their evening or night shifts compared to day shifts. In contrast, their share of energy from snacking in the evening or night shift was significantly higher when compared to their day shift counterparts. Nurses consumed fewer meals and demonstrated more frequent snacking on evening and night shifts. This suggests that it is possible that shift nurses consume more sugary, high-fat, and fast meals and snacks during night duty to stay awake and recover from the heavy workload and long shift [57].

In this study, there was a high processed meat content in the diets of the midwives surveyed. Similar results were obtained in a study on the diet and nutritional status of the Polish population. Almost forty percent (39.2%) of women reported consuming high-quality cold meats 2–3 times a week. The consumption frequency of 4–5 times a week was declared by 23% of women. The International Agency for Research on Cancer of the World Health Organisation has indicated that the consumption of red meat is probably carcinogenic to humans, while processed meat is considered carcinogenic [46]. The World Cancer Research Fund/American Institute for Cancer Research recommends limiting red meat consumption to moderate amounts and eating very little processed meat [58].

The mean BMI of the studied midwives was greater than 25 kg/m^2^, which indicates the presence of overweight. In a study by Livingstone et al. [59], a higher HDI value was associated with a lower body fat percentage, lower trunk fatness and lean body mass, and higher bone mineral content. In that study, there was a benefit of higher diet quality in reducing total fat mass. It was most pronounced in those with a higher overall risk of obesity. On the other hand, in our study, it was found that women with a body fat percentage of ≤30% had significantly lower BMI values but obtained significantly higher percentages of energy from sweets consumption compared to women with a higher body fat percentage. In a study by Shaw et al. [14], snacking during the night shift was found to have a lower energy value on average, while overall total sugar intake was higher. The lower energy supply despite the high proportion of sweets may explain the lower body weight of the midwives in this study. However, the consumption of meals at times of day that are associated with reduced glucose tolerance [60], especially those with high sugar content, may be unfavorable.

The authors observed that more than half of the women chose sweets as an ingredient in their diet on a daily basis, and 44% of the participants used sugar for sweetening beverages on a daily basis. These results are consistent with the declared consumption of sugar and sweets in the Polish population [55]. In a study by Shaw et al. [14], which analyzed the dietary patterns of shift workers, it was found that the % of E from total sugar intake during the night shift was higher compared to the day shift/non-working day, while a higher saturated fat intake was reported during the periods of the day shift/non-working day compared to the periods of the night shift. Shaw et al. [14], as the authors of this study concluded, found that interventions to improve dietary choices should include education on proper menu composition on all days and not focus only on the periods during a night shift.

In our study, we assessed the quality of the diet of only Polish (Wrocław) midwives working in shifts. Unhealthy nutritional habits impacting the diet quality of healthcare shift workers have also been demonstrated in studies, including the populations of Turkey [61], Iran [49], the Netherlands [62,63], Taiwan [11], and Australia [64]. Representatives of non-medical professions working in shifts, e.g., firefighters [65], manufacturing workers, shift leaders or office workers [66], and other occupations [67] are also characterized by poor diet quality and bad nutritional habits.

The results of the study among one occupation of healthcare workers coincided with the results of studies including some health professionals (e.g., from Poland [68] and Greece [69]) or incorporated nurses and midwives in one group (Poland [51], Australia [53,70], New Zealand, United Kingdom [70]). Also, the result can reflect studies of nurse populations, including nurse–midwives, in countries where midwives are also nurse practitioners. The results point out that low diet quality does not depend on graduating from specific study courses in healthcare areas or working selected occupations (midwives). Although the specificity of dietary choices seems to differ between professional groups and may be determined by gender [63], the problem of properly balancing the diet among employees working night shifts seems to be universal, both in Poland and in other countries. This indicates that systemic solutions covering healthcare workers with shift work developed globally may successfully also have a positive impact on this professional group.

Although midwives’ diets are of poor nutritional quality, studies involving nutrition training programs for nurses have shown that they can be effective in helping to increase nutritional knowledge and awareness [54]. Due to the important role of midwives in educating women in the perinatal period, their greater knowledge about proper nutrition and its influence on health may have a positive impact on other population groups with whom they have contact in hospital wards [15].

### Limitations

This study is a cross-sectional study with some limitations that should be mentioned. The methodology was designed to obtain a highly homogeneous group of participants representing one distinctive occupation (midwives) and workplace (only public hospitals in Wrocław). Therefore, the study group was not combined with other medical professionals, e.g., nurses or doctors, as was practiced in other studies [51,68,70]. These assumptions contributed to the unification of the group but limited its size.

Moreover, due to the perceived high work intensity and low motivation to take part in the survey, some women did not agree to participate or provide an incomplete food diary (*n* = 27), highlighting that it was too time consuming. The authors are aware that an increasing the number of participants would improve the credibility of the obtained results, and its current size (*n* = 50) is the main limitation.

Specification of midwives’ work, such as high intensity, irregular day rhythm, unpredictability number, and duration of breaks, was the main reason for establishing a self-administrated 3-day food diary as a recorded method in this study due to relatively low levels of difficulty in supplementation and easy access. However, it creates possible limitations, such as over-/under-estimation of portions, and incomplete records of consumed meals, beverages, or products. Due to the mentioned limitation, we are aware that the addition of other more advanced methods, such as photo-assisted recall or the double weighting method, could increase the clarification of the obtained results and increase their credibility. However, in the current study group, the use of, for example, the double weighing method requiring appropriate storage and collection of samples, increasing the effort of the subjects, would additionally limit their number. Furthermore, AHEI-2015 values were estimated in this study using a 3-day food diary, excluding the FFQ. This may reduce the precision of estimated portions; however, it is acceptable to use it [37].

## 5. Conclusions

The midwives’ diets had low adherence to the analyzed DQIs. Therefore, it seems necessary to educate, or in some cases re-educate, shift midwives regarding healthy food product choices. Moreover, psychological tools to support these choices and improve self-stress management to minimize the risk of emotional eating are needed. It seems that educational programs should provide group training (work–society influence) and individual consultation with dietitians to focus on easy solutions for the routine consumption of sweets, processed meat, and refined bakery and confectionery products. Also, it could provide some useful, simple, and self-administered tools to create healthy menus or meals during shift work; for example, a scheme based on DASH diet guidelines. To enable midwives to change their eating habits, it is also necessary to implement systemic solutions, like access to healthy meals and snacks in the workplace. The presence of high-quality products in hospital shops and vending machines and ensuring the possibility of eating nutritious meals during night working hours would undoubtedly support making healthy food choices.

The proposed solutions, due to the universality of the problem of low-quality diets among shift workers and the risk of metabolic disorders resulting from working at hours that disturb the circadian cycle, may constitute the basis for developing recommendations for education and nutritional care of representatives of various groups of shift workers. The above-mentioned changes, by improving the quality of the diet of midwives working shifts, will have a positive impact on their body mass and metabolic health, reducing the risk of cardiovascular diseases and diabetes. A balanced diet will also support the effectiveness of midwives’ work—their focus and attention on the work they perform and their alertness to unexpected situations. This, in turn, will directly affect the quality of services provided by healthcare and thus their reception by the patient.

## Figures and Tables

**Table 5:** The percentage of diets with an appropriate degree of adherence to the dietary pattern described by the HDI-2015.

Index	Low Adherence	Medium Adherence	High Adherence	$\bar{X}$ ± SD (*n* = 50)
HDI 2015	66%	30%	4%	2.94 ± 1.67

**Table 6:** The percentage of diets simultaneously achieved a low or higher than low adherence to a selected amount of dietary patterns.

Adherence Level	Low Adherence	Adherence Higher than Low	
Selected Indices		Moderate or Higher Adherence	Good or High Adherence
**4 indices**—AHEI-2010, HDI-2015, HEI-2015, and Mellen’s DASH diet index	96%	4%	0%
**3 indices**—Mellen’s DASH diet index, HDI-2015, and HEI-2015	84%	16%	4%
**2 indices**—Mellen’s DASH diet and HDI-2015	80%	20%	8%
**2 indices**—Mellen’s DASH diet and HEI-2015	78%	22%	8%
**2 indices**—HDI-2015 and HEI-2015	78%	22%	4%

**Table 7:** A comparison of the values of anthropometric measurements and dietary components of the participants whose diets scored ≥4.5 points (*n* = 30) and <4.5 points (*n* = 20) on Mellen’s DASH diet index.

	Mellen’s DASH Diet Score ≥4.5 (*n* = 30)		Mellen’s DASH Diet Score <4.5 (*n* = 20)		*p*
	$\bar{X}$ ± SD	M	$\bar{X}$ ± SD	M	
**Anthropometric variables**					
*Age* [years]	45.75 ± 10.27	48.50	45.97 ± 11.39	49.00	0.968
Body weight [kg]	74.21 ± 15.36	76.30	68.66 ± 13.14	68.55	0.184
BMI [kg/m^2^] (ref. 18.5 to 24.9)	27.92 ± 5.93	27.94	26.07 ± 4.92	24.82	0.243
Waist circumference [cm] (ref. <80 cm)	88.10 ± 15.49	87.50	83.23 ± 10.92	83.00	0.205
Body fat [%] (ref. 20–30%)	34.69 ± 9.03	35.85	32.55 ± 7.15	33.20	0.363
Visceral fat level (ref. 0–14)	7.1 ± 3.60	7.50	6.13 ± 3.00	7.00	0.315
**Diet components**					
Processed meat [g]	**35.92 ± 29.68**	36.67	**64.79 ± 42.90**	55.00	0.012 *
*Added sugar* [g]	3.72 ± 8.95	**0.00**	12.43 ± 11.40	**10.00**	<0.001 *
*Sweet beverages* [mL]	51.67 ± 128.12	0.00	36.67 ± 90.80	0.00	0.938
Number of meals	**4.52 ± 0.68**	4.33	**4.96 ± 0.71**	5.00	0.036 *
*Total energy intake* [kcal]	1568.25 ± 377.05	**1489.47**	2072.79 ± 469.19	**2068.96**	<0.001 *
Energy intake from breakfast [kcal]	**246.61 ± 107.69**	245.81	**318.64 ± 117.84**	339.98	0.035 *
% of E from breakfast [%]	16.30 ± 6.59	15.52	15.97 ± 5.49	16.33	0.851
*% of E from sweets* [%]	7.01 ± 6.64	**6.17**	14.41 ± 11.89	**12.51**	0.027 *
*Energy intake from snacking* [kcal]	222.15 ± 201.58	165.08	272.02 ± 273.43	219.47	0.579
*% of E from snacking* [%]	12.97 ± 8.56	11.41	12.32 ± 11.15	11.38	0.649

Parameters with non-normally distributed data are indicated in italics. The median should be described to compare their values.* Statistically significant differences—*p* < 0.05.

**Table 8:** A comparison of the values of anthropometric measurements and dietary components in participants’ diets in which they obtained ≤ 13% and >13% of E in terms of snacking between meals.

	% of E Obtained from Snacking ≤ 13% (*n* = 30)		% of E Obtained from Snacking > 13% (*n* = 20)		*p*
	$\bar{X}$ ± SD	M	$\bar{X}$ ± SD	M	
**Anthropometric variables**					
Body weight [kg]	71.18 ± 13.96	70.35	70.43 ± 14.57	68.55	0.858
BMI [kg/m^2^] (ref. 18.5 to 24.9)	26.71 ± 5.09	25.82	26.96 ± 5.78	26.14	0.876
Waist circumference [cm] (ref. <80 cm)	85.47 ± 12.22	84.50	84.75 ± 14.23	86.50	0.853
Body fat [%] (ref. 20–30%)	33.55 ± 7.54	35.35	33.20 ± 8.55	32.85	0.880
Visceral fat level (ref. 0–14)	6.60 ± 3.12	7.00	6.40 ± 3.47	6.00	0.836
**Diet components**					
*Processed meat* [g]	52.23 ± 40.45	42.50	54.75 ± 39.75	45.00	0.804
*Added sugar* [g]	6.77 ± 9.87	0.83	12.20 ± 12.26	8.33	0.102
*Sweet beverages* [mL]	15.56 ± 49.05	0.00	83.33 ± 148.79	0.00	0.154
Number of meals	4.9 ± 0.63	5.00	4.6 ± 0.81	4.33	0.157
Total energy intake [kcal]	**1734.29 ± 462.59**	1634.37	**2076.00 ± 472.30**	2120.61	0.016 *
Energy intake from breakfast [kcal]	267.20 ± 123.65	283.87	323.78 ± 99.96	344.59	0.101
*% of E from breakfast* [%]	16.06 ± 6.82	16.46	16.16 ± 4.18	15.25	0.656
*% of E from sweets* [%]	10.79 ± 8.86	8.34	12.44 ± 12.75	8.79	0.984
*Energy intake from snacking* [kcal]	104.45 ± 83.61	**73.29**	473.50 ± 241.52	**403.57**	<0.001 *
*% of E from snacking* [%]	6.10 ± 4.72	**5.12**	22.30 ± 7.94	**21.23**	<0.001 *

Parameters with non-normally distributed data are indicated in italics. The median should be described to compare their values. * Statistically significant differences—*p* < 0.05.

**Table 9:** A comparison of the values of anthropometric measurements and dietary components in participants’ diets in which they obtained ≤12% and >12% of their daily energy intake from consuming sweets.

	% of E obtained from Sweets Consumption ≤12% (*n* = 30)		% of E obtained from Sweet Consumption > 12% (*n* = 20)		*p*
	$\bar{X}$ ± SD	M	$\bar{X}$ ± SD	M	
**Anthropometric variables**					
Body weight [kg]	73.76 ± 13.36	73.05	66.56 ± 14.35	63.30	0.082
BMI [kg/m^2^] (ref. 18.5 to 24.9)	27.78 ± 5.21	27.17	25.35 ± 5.29	23.64	0.122
Waist circumference [cm] (ref. <80 cm)	**88.43 ± 12.66**	88.50	**80.30 ± 12.11**	77.00	0.031 *
Body fat [%] (ref. 20–30%)	**35.47 ± 7.39**	36.45	**30.31 ± 7.77**	30.00	0.024 *
Visceral fat level (ref. 0–14)	7.03 ± 3.28	7.00	5.75 ± 3.08	6.50	0.180
**Diet components**					
*Processed meat* [g]	49.23 ± 40.93	43.33	59.25 ± 38.42	48.33	0.400
*Added sugar* [g]	8.88 ± 12.59	2.17	9.03 ± 8.74	8.33	0.501
*Sweet beverages* [mL]	50.56 ± 117.65	0.00	30.83 ± 86.65	0.00	0.526
*Number of meals*	4.78 ± 0.68	4.67	4.78 ± 0.78	5.00	0.827
Total energy intake [kcal]	**1716.42 ± 411.03**	1609.30	**2102.81 ± 520.68**	2112.26	0.006 *
Energy intake from breakfast [kcal]	283.06 ± 107.50	282.13	299.98 ± 131.68	317.95	0.628
*% of E from breakfast* [%]	17.19 ± 6.30	16.46	14.46 ± 4.82	15.24	0.151
*% of E from sweets* [%]	4.51 ± 3.91	**5.36**	21.86 ± 8.85	**19.28**	<0.001 *
*Energy intake from snacking* [kcal]	240.22 ± 221.79	199.99	269.85 ± 276.09	181.28	0.913
*% of E from snacking* [%]	13.00 ± 9.13	12.40	11.94 ± 11.31	9.86	0.559

Parameters with non-normally distributed data are indicated in italics. The median should be described to compare their values; * statistically significant differences—*p* < 0.05.

**Table 10:** A comparison of dietary components in the group of participants marked by body fat content ≤ 30% (*n* = 15) and >30% (*n* = 35).

	Diets of Participants with Body fat ≤30% (*n* = 15)		Diets of Participants with Body fat >30% (*n* = 35)		*p*
	$\bar{X}$ ± SD	M	$\bar{X}$ ± SD	M	
**Diet components**					
*Processed meat* [g]	67.69 ± 48.43	73.33	47.05 ± 34.36	43.33	0.193
*Added sugar* [g]	11.22 ± 12.06	10.00	7.97 ± 10.67	3.33	0.567
*Sweet beverages* [mL]	16.67 ± 33.88	0.00	53.81 ± 124.03	0.00	0.766
*Number of meals*	4.73 ± 0.80	5.00	4.80 ± 0.69	4.67	0.799
*Total energy intake* [kcal]	2002.25 ± 488.34	2085.28	1814.71 ± 488.02	1655.66	0.244
*Energy intake from breakfast* [kcal]	324.39 ± 98.46	341.55	275.02 ± 122.58	275.57	0.271
*% of E from sweets* [%]	16.60 ± 11.65	**16.33**	9.24 ± 9.31	**6.22**	0.027 *
*Energy intake from snacking* [kcal]	236.80 ± 197.80	192.23	258.62 ± 262.89	193.32	0.941
*% of E from snacking* [%]	11.31 ± 8.87	9.15	13.12 ± 10.50	12.22	0.743

Parameters with non-normally distributed data are indicated in italics. The median should be described to compare their values. * Statistically significant differences—*p* < 0.05.

**Table 11:** A comparison of dietary components in the group of participants with waist circumference values ≤ 80 cm (*n* = 18) and >80 cm (*n* = 32).

	Diets of Participants with Waist Circumference Values ≤ 80 cm (*n* = 18)		Diets of Participants with Waist Circumference Values > 80 cm (*n* = 32)		*p*
	$\bar{X}$ ± SD	M	$\bar{X}$ ± SD	M	
**Diet components**					
*Processed meat* [g]	53.94 ± 47.46	38.33	52.82 ± 35.54	45.00	0.785
*Added sugar* [g]	11.39 ± 11.71	10.00	7.57 ± 10.67	3.00	0.419
*Sweet beverages* [mL]	28.70 ± 79.31	0.00	50.52 ± 118.77	0.00	0.635
*Number of meals*	4.7 ± 0.80	4.67	4.82 ± 0.67	4.67	0.578
*Total energy intake* [kcal]	2093.97 ± 485.04	**2093.45**	1745.54 ± 455.82	**1616.56**	0.025 *
Energy intake from breakfast [kcal]	311.86 ± 115.34	340.67	277.44 ± 117.76	272.45	0.317
*% of E from sweets* [%]	17.10 ± 10.91	**16.92**	8.27 ± 9.01	**6.17**	0.004 *
*Energy intake from snacking* [kcal]	274.10 ± 242.37	221.23	239.68 ± 246.20	189.87	0.642
*% of E from snacking* [%]	12.55 ± 9.96	9.90	12.59 ± 10.14	12.13	0.935

Parameters with non-normally distributed data are indicated in italics. The median should be described to compare their values. * Statistically significant differences—*p* < 0.05.

**Table 12:** Mean content of selected food items in the diets of the surveyed women (*n* = 50).

Food Items	$\bar{X}$ ± SD	MIN	MAX
Added sugar [g]	9.00 ± 13.10	0.00	55.00
Sweet beverages [mL]	41.28 ± 158.40	0.00	1100.00
Sweets and cakes [g]	70.60 ± 108.83	0.00	720.00
Caffeine [mg]	147.27 ± 100.56	0.00	534.20
Processed meat [g]	52.96 ± 59.14	0.00	255.00
Vegetables [g]	442.50 ± 234.99	0.00	1675.00
Fruits [g]	250.54 ± 233.11	0.00	1260.00
Fish [g]	14.88 ± 42.09	0.00	200.00
Eggs [g]	26.31 ± 45.61	0.00	180.00
Nuts and seeds [g]	6.88 ± 21.54	0.00	175.00
Natural dairy [g]	185.41 ± 165.54	0.00	730.00
Legumens [g]	4.49 ± 18.14	0.00	100.00

## Data Availability

All the data used in this study are available, and the lead author has full access to the data reported in the manuscript.
